# DrosoPhyla: Resources for Drosophilid Phylogeny and Systematics

**DOI:** 10.1093/gbe/evab179

**Published:** 2021-08-03

**Authors:** Cédric Finet, Victoria A Kassner, Antonio B Carvalho, Henry Chung, Jonathan P Day, Stephanie Day, Emily K Delaney, Francine C De Ré, Héloïse D Dufour, Eduardo Dupim, Hiroyuki F Izumitani, Thaísa B Gautério, Jessa Justen, Toru Katoh, Artyom Kopp, Shigeyuki Koshikawa, Ben Longdon, Elgion L Loreto, Maria D S Nunes, Komal K B Raja, Mark Rebeiz, Michael G Ritchie, Gayane Saakyan, Tanya Sneddon, Machiko Teramoto, Venera Tyukmaeva, Thyago Vanderlinde, Emily E Wey, Thomas Werner, Thomas M Williams, Lizandra J Robe, Masanori J Toda, Ferdinand Marlétaz

**Affiliations:** 1 Howard Hughes Medical Institute and Laboratory of Molecular Biology, University of Wisconsin, Madison, USA; 2 Departamento de Genética, Instituto de Biologia, Universidade Federal do Rio de Janeiro, Brazil; 3 Department of Entomology, Michigan State University, USA; 4 Department of Genetics, University of Cambridge, United Kingdom; 5 Department of Biological Sciences, University of Pittsburgh, USA; 6 Department of Evolution and Ecology, University of California-Davis, USA; 7 Programa de Pós-Graduação em Biodiversidade Animal, Universidade Federal de Santa Maria, Rio Grande do Sul, Brazil; 8 Department of Biological Sciences, Faculty of Science, Hokkaido University, Sapporo, Japan; 9 Programa de Pós-Graduação em Biologia de Ambientes Aquáticos Continentais, Universidade Federal do Rio Grande, Rio Grande do Sul, Brazil; 10 The Hakubi Center for Advanced Research and Graduate School of Science, Kyoto University, Japan; 11 Centre for Ecology and Conservation, College of Life and Environmental Sciences, University of Exeter, Exeter, United Kingdom; 12 Department of Biological and Medical Sciences, Oxford Brookes University, United Kingdom; 13 Centre for Functional Genomics, Oxford Brookes University, United Kingdom; 14 Department of Biological Sciences, Michigan Technological University, USA; 15 School of Biology, University of St Andrews, United Kingdom; 16 Department of Biology, University of Dayton, USA; 17 Hokkaido University Museum, Hokkaido University, Sapporo, Japan; 18 Centre for Life’s Origins and Evolution, Department of Genetics, Evolution and Environment, University College London, United Kingdom

**Keywords:** Drosophilidae, phylogenomics, systematics

## Abstract

The vinegar fly *Drosophila melanogaster* is a pivotal model for invertebrate development, genetics, physiology, neuroscience, and disease. The whole family Drosophilidae, which contains over 4,400 species, offers a plethora of cases for comparative and evolutionary studies. Despite a long history of phylogenetic inference, many relationships remain unresolved among the genera, subgenera, and species groups in the Drosophilidae. To clarify these relationships, we first developed a set of new genomic markers and assembled a multilocus data set of 17 genes from 704 species of Drosophilidae. We then inferred a species tree with highly supported groups for this family. Additionally, we were able to determine the phylogenetic position of some previously unplaced species. These results establish a new framework for investigating the evolution of traits in fruit flies, as well as valuable resources for systematics.

## Introduction

The vinegar fly *Drosophila melanogaster* is a well-established and versatile model system in biology (Hales et al. 2015). The story began at the start of the 20th century when the entomologist Charles Woodworth bred *D. melanogaster* in captivity, paving the way to William Castle’s seminal work at Harvard in 1901 (Sturtevant 1959). But it is undoubtedly with Thomas Hunt Morgan and his colleagues that *D. melanogaster* became a model organism in genetics (Morgan 1910). Nowadays, *D. melanogaster* research encompasses diverse fields, such as biomedicine (Ugur et al. 2016), developmental biology (Hales et al. 2015), growth control (Wartlick et al. 2011), gut microbiota (Trinder et al. 2017), innate immunity (Buchon et al. 2014), behavior (Cobb 2007), and neuroscience (Bellen et al. 2010).

By the mid-20th century, evolutionary biologists have widened *Drosophila* research by introducing many new species of Drosophilidae in comparative studies. For example, the mechanisms responsible for morphological differences of larval denticle trichomes (Sucena et al. 2003; McGregor et al. 2007), adult pigmentation (Jeong et al. 2008; Yassin, Delaney, et al. 2016), sex combs (Tanaka et al. 2009), and genital shape (Glassford et al. 2015; Peluffo et al. 2015) have been thoroughly investigated across Drosophilidae. Comparative studies brought new insights into the evolution of ecological traits, such as host specialization (Lang et al. 2012; Yassin et al. 2016), niche diversification (Chung et al. 2014), species distribution (Kellermann et al. 2009), pathogen virulence (Longdon et al. 2015), and behavior (Dai et al. 2008; Karageorgi et al. 2017).

More than 150 genomes of *Drosophila* species are now sequenced (Adams et al. 2000; Clark et al. 2007; Wiegmann and Richards 2018; Kim et al. 2021), allowing the comparative investigation of gene families (Sackton et al. 2007; Almeida et al. 2014; Finet et al. 2019) as well as global comparison of genome organization (Bosco et al. 2007; Bhutkar et al. 2008). For all these studies, a clear understanding of the historical relationships between species is necessary to interpret the results in an evolutionary context. A robust phylogeny is then crucial to confidently infer ancestral states, identify synapomorphic traits, and reconstruct the history of events during the evolution and diversification of Drosophilidae.

Fossil-based divergence time estimation suggest that the family Drosophilidae originated at least 30–50 Ma (Throckmorton 1975; Grimaldi 1987; Wiegmann et al. 2011). To date, the family comprises more than 4,400 species (DrosWLD-Species 2021; Available from: https://bioinfo.museum.hokudai.ac.jp/db/index.php; last accessed June 29, 2021) classified into two subfamilies, the Drosophilinae Rondani and the Steganinae Hendel. Each of these subfamilies contains several genera, which are traditionally subdivided into subgenera, and are further composed of species groups. Nevertheless, the monophyletic status of each of these taxonomic units is frequently controversial or unassessed. Part of this controversy is related to the frequent detection of paraphyletic taxa within Drosophilidae (Throckmorton 1975; Katoh et al. 2000, 2017; Robe et al. 2005; Da Lage et al. 2007; Robe, Loreto, et al. 2010; Van Der Linde et al. 2010; Russo et al. 2013; Yassin 2013; Gautério et al. 2020), although the absence of a consistent phylogenetic framework for the entire family makes it difficult to assess alternative scenarios.

Despite the emergence of the *Drosophila* genus as a model system to investigate the molecular genetics of functional evolution, relationships within the family Drosophilidae remain poorly supported. The first modern phylogenetic trees of this family relied on morphological characters (Throckmorton 1962, 1975, 1982), followed by a considerable number of molecular phylogenies that mainly focused on individual species groups (reviewed in Markow and O’Grady [2006], O’Grady and DeSalle [2018]). For the last decade, only a few large-scale studies have attempted to resolve the relationships within Drosophilidae as a whole. For example, supermatrix approaches brought new insights, such as the identification of the earliest branches in the subfamily Drosophilinae (Van Der Linde et al. 2010; Yassinet al. 2010), the paraphyly of the subgenus *Drosophila* (*Sophophora*) (Gao et al. 2011), the placement of Hawaiian clades (O’Grady et al. 2011; Lapoint et al. 2013; Katoh et al. 2017), and the placement of Neotropical Drosophilidae (Robe et al. 2010). Most of the aforementioned studies have suffered from limited taxon or gene sampling. Recent studies improved the taxon sampling and the number of loci analyzed (Morales-Hojas and Vieira 2012; Russo et al. 2013; Izumitani et al. 2016). To date, the most taxonomically broad study is a revision of the Drosophilidae that includes 30 genera in Steganinae and 43 in Drosophilinae, but only considering a limited number of genomic markers (Yassin 2013).

To clarify the phylogenetic relationships in the Drosophilidae, we built a comprehensive data set of 704 species that include representatives from most of the major genera, subgenera, and species groups in this family. We developed new genomic markers and compiled available ones from previously published phylogenetic studies. We then inferred well-supported trees at the group- and species-level for this family. Additionally, we were able to determine the phylogenetic position of several species of uncertain affinities. Our results establish a new framework for investigating the systematics and diversification of fruit flies and provide a valuable genomic resource for the *Drosophila* community.

## Results and Discussion

### A Multigene Phylogeny of 704 Drosophilid Species

**Fig. 1.**
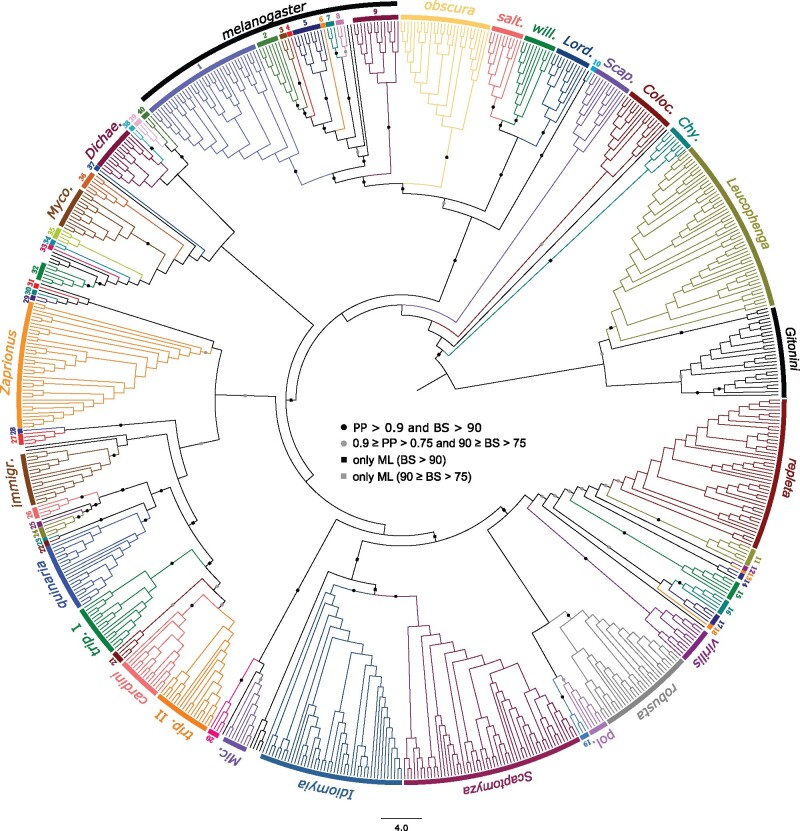
Phylogram of the 704-taxon analyses. IQ-TREE maximum-likelihood analysis was conducted under the GTR+R+FO model. Support values obtained after 100 bootstrap replicates are shown for selected supragroup branches, and infragroup branches within the *melanogaster* group (all the support values are shown online). Black dots indicate support values of PP>0.9 and BP>90; gray dots 0.9 ≥ PP>0.75 and 90 ≥ BP>75; black squares only BP>90; gray squares only 90 ≥ BP>75. Scale bar indicates the number of changes per site. Groups and subgroups are numbered or abbreviated as follows: (1) *montium*, (2) *takahashii* sgr, (3) *suzukii* sgr, (4) *eugracilis* sgr, (5) *melanogaster* sgr, (6) *ficusphila* sgr, (7) *elegans* sgr, (8) *rhopaloa* sgr, (9) *ananassae*, (10) *Collessia*, (11) *mesophragmatica*, (12) *dreyfusi*, (13), *coffeata*, (14) *canalinea*, (15) *nannoptera*, (16) *annulimana*, (17) *flavopilosa*, (18) *flexa*, (19) *angor*, (20) *Dorsilopha*, (21) *ornatifrons*, (22) *histrio*, (23) *macroptera*, (24) *testacea*, (25) *bizonata*, (26) *funebris*, (27) *Samoaia*, (28) *quadrilineata* sgr, (29) *Liodrosophila*, (30) *Hypselothyrea*, (31) *Sphaerogastrella*, (32) *Zygothrica* I, (33) *Paramycodrosophila*, (34) *Hirtodrosophila* III, (35) *Hirtodrosophila* II, (36) *Hirtodrosophila* I, (37) *Dettopsomyia*, (38) *Mulgravea*, (39) *Hirtodrosophila* IV, (40) *Zygothrica* II, *Chy*, *Chymomyza*; *Colo*, *Colocasiomyia*; *Dichae*, *Dichaetophora*; *immigr*, *immigrans*; *Lord*, *Lordiphosa*; *Mic*, *Microdrosophila*; *Myco*, *Mycodrosophila*; *pol*, *polychaeta*; *salt*, *saltans*; *Scap*, *Scaptodrosophila*; *trip*, *tripunctata*; *will*, *willistoni*.

**Fig. 2.**
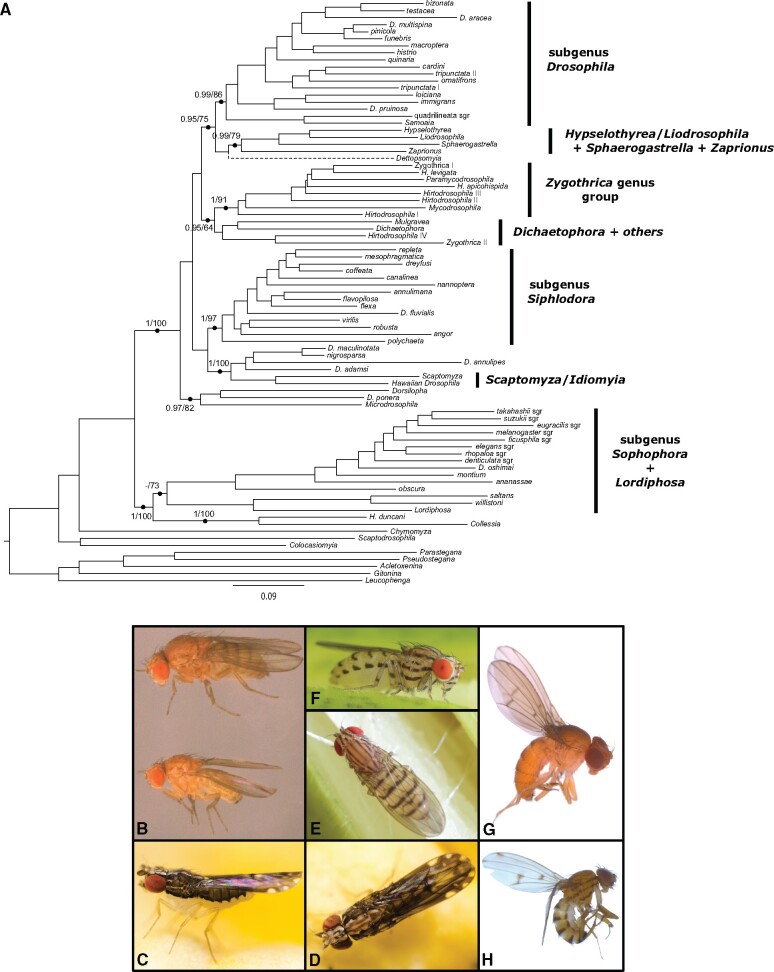
(*A*) Phylogram of the 83-taxon analyses. The overall matrix represents 14,961 nucleotides and 83 taxa, including 63 composite ones. Support values obtained after 100 bootstrap replicates and Bayesian posterior probabilities are shown for selected branches and mapped onto the ML topology (all the support values are shown in supplementary fig. S1, Supplementary Material online). The dotted line indicates that the placement of *Dettopsomyia* varies between ML and Bayesian trees. Scale bar indicates the number of changes per site. (*B–H*) Photos of species of particular interest in this article. (*B*) *Drosophila oshimai* female (top) and male (bottom) (Japan, courtesy of Japan Drosophila Database), (*C* and *D*) *Collessia kirishimana* (Japan, courtesy of Masafumi Inoue), (*E* and *F*) *Drosophila annulipes* (Japan, courtesy of Yasuo Hoshino), (*G*) *Drosophila pruinosa* (São Tomé, courtesy of Stéphane Prigent), (*H*) *Drosophila adamsi* (Cameroun, courtesy of Stéphane Prigent).

We assembled a multilocus data set of 17 genes (14,961 unambiguously aligned nucleotide positions) from 704 species of Drosophilidae. Our phylogeny recovers many of the clades or monophyletic groups previously described in the Drosophilidae (fig. 1). Although the branching of the species groups is generally well-supported, we observe that some of the deepest branches of the phylogenic tree remain poorly supported or unresolved, especially in Bayesian analyses (supplementary figs. S1 and S2, Supplementary Material online). This observation prompted us to apply a composite taxon strategy that has been used to resolve challenging phylogenetic relationships (Finet et al. 2010; Campbell and Lapointe 2011; Sigurdsen and Green 2011; Charbonnier et al. 2015; Mengual et al. 2017; Fan et al. 2020). This approach limits branch lengths in selecting slow-evolving sequences, and decreases the percentage of missing data, improving phylogenetic reconstruction for sparse data matrices (Campbell and Lapointe 2009). We defined 63 composite groups as the monophyletic groups identified in the 704-taxon analysis (fig. 1 and supplementary table S1, Supplementary Material online), and added these to the sequences of 20 other ungrouped taxa to perform additional phylogenetic evaluations. The overall bootstrap values and posterior probabilities were higher for the composite tree (fig. 2*A* and supplementary figs. S3 and S4, Supplementary Material online). In addition, we applied the summary method ASTRAL to our composite data set to infer a species tree from a collection of input trees. However, the resulting tree is less resolved than the one obtained by concatenation (supplementary fig. S5, Supplementary Material online).

**Fig. 3.**
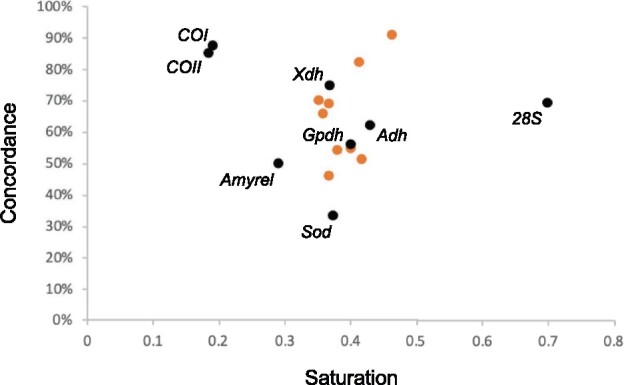
Concordance versus mutational saturation of the phylogenetic markers. The *y* axis indicates the percentage of concordant nodes, and the *x* axis indicates the saturation level. In comparison with published markers (black dots), the markers developed in this study (orange dots) generally show moderate saturation levels and satisfying concordance.

Incongruence among phylogenetic markers can be related to incomplete lineage sorting, introgression, hybridization, or other processes and can be detrimental to accurate species tree reconstruction (Jeffroy et al. 2006; Kapli et al. 2020). In order to estimate the presence of incongruent signal in our data set, we first investigated the qualitative effect of single marker removal on the topology of the composite tree (supplementary fig. S6, Supplementary Material online). We found the overall topology is very robust to marker sampling, with only a few minor changes for each data set. For instance, the *melanogaster* subgroup sometimes clusters with the *eugracilis* subgroup instead of branching off prior to the *eugracilis* subgroup (fig. 2 and supplementary fig. S6, Supplementary Material online). The position of the genus *Dettopsomyia* and that of the *angor* and *histrio* groups is also very sensitive to single marker removal, which could explain the low support values obtained (fig. 2 and supplementary fig. S6, Supplementary Material online). To a lesser extent, the position of *Drosophila fluvialis* can vary as well depending on the removed marker (fig. 2 and supplementary fig. S6, Supplementary Material online). We also quantitatively investigated the incongruence present in our data set by calculating genealogical concordance. The gene concordance factor is defined as the percentage of individual gene trees containing that node for every node of the reference tree. Similarly, the fraction of nodes supported by each marker can be determined. The markers we developed in this study show concordance rates ranging from 46.2% to 90.9% (fig. 3 and table 1). With an average concordance rate of 65%, these new markers appear as credible phylogenetic markers, without significantly improving the previous markers (average concordance rate of 64.8%).

**Table 1:** Data Set Statistics

Name	No. Sequences	No. Sites	Informative Sites (%)	Inferred Distance	Observed Distance	Saturation	No. Concording Nodes	No. Missing Nodes	Concordance (%)
*28S*	49/83	848	18.4	0.200	0.189	0.700	25/80	44	69.4
*Adh*	53/83	724	54.4	0.886	0.331	0.430	28/80	35	62.2
*Amyrel*	48/83	1475	53.5	2.458	0.545	0.290	18/80	44	50.0
*COI*	51/83	1438	33.8	1.119	0.666	0.191	35/80	40	87.5
*COII*	57/83	688	37.8	1.004	0.169	0.185	40/80	33	85.1
*Gpdh*	26/83	859	35.0	0.784	0.286	0.400	9/80	64	56.3
*Sod*	22/83	574	49.3	1.072	0.333	0.373	4/80	68	33.3
*Xdh*	19/83	2088	42.4	0.919	0.314	0.368	9/80	68	75.0
									
*Ddc*	52/83	1162	42.3	1.003	0.262	0.358	27/80	39	65.9
*Dll*	56/83	377	30.8	0.629	0.229	0.463	40/80	36	90.9
*eb*	67/83	891	46.7	1.247	0.318	0.380	32/80	21	54.2
*en*	51/83	1119	51.1	1.009	0.307	0.371	18/80	41	46.2
*eve*	66/83	806	48.6	1.083	0.303	0.367	40/80	22	69.0
*hh*	63/83	486	62.6	1.203	0.352	0.400	29/80	27	54.7
*Notum*	51/83	672	62.6	1.005	0.352	0.417	18/80	45	51.4
*ptc*	60/83	430	55.8	1.076	0.323	0.413	42/80	29	82.4
*wg*	57/83	324	51.5	1.223	0.321	0.352	33/80	33	70.2

Multiple substitutions at the same position is another classical bias in phylogenetic reconstruction, capable of obscuring the genuine phylogenetic signal (Jeffroy et al. 2006). We quantified the mutational saturation for each phylogenetic marker. On an average, the newly developed markers are moderately saturated (fig. 3, supplementary fig. S7, Supplementary Material online, and table 1). These markers are indeed less saturated than the *Amyrel*, *COI*, and *COII* genes that have been commonly applied for phylogenetic inference in Drosophilidae (Baker and Desalle 1997; O’Grady et al. 1998, 2011; Remsen and O’Grady 2002; Bonacum et al. 2005; Da Lage et al. 2007; Robe et al. 2010; Gao et al. 2011; Russo et al. 2013; Yassin 2013).

In the following sections of the article, we will highlight and discuss some of the most interesting results we obtained. Our analyses either confirm or challenge previous phylogenies and shed light on several unassessed questions, contributing to an emerging picture of phylogenetic relationships in Drosophilidae.

### The Steganinae Subfamily

To avoid long-branch attraction due to some divergent steganine sequences, we compiled a more specific and comprehensive data set from 164 taxa of Steganinae (vs. 80 taxa in the 704-taxon analysis). Whereas morphology-based studies suggest the monophyly of Steganinae (Okada 1989; Grimaldi 1990), molecular phylogenetic have led to contradictory results (Remsen and O’Grady 2002; Otranto et al. 2008; Van Der Linde et al. 2010; Russo et al. 2013; Yassin 2013). Our study identifies the Steganinae as monophyletic for both data sets (fig. 1 and supplementary fig. S8, Supplementary Material online) and supports a recent phylogenomic study of Steganinae (Dias et al. 2020). The topology within the Steganinae substantially differs from the division of the subfamily into two monophyletic tribes: Steganini and Gitonini (Yassin 2013). Our study does not recover the monophyly of the genera *Leucophenga* and *Parastegana*, only due to the placement of the two species *Leucophenga maculata* and *Parastegana femorata*. Future studies are needed to disentangle possible contamination and true phylogenetic position. We also found the branching of some *Colocasiomyia* species within the Steganinae (supplementary fig. S8, Supplementary Material online). This finding, which challenges previous published cladograms of *Colocasiomyia* (Grimaldi 1991; Sultana et al. 2006) and our 704-taxon analysis (fig. 1), is likely an artifact of reconstruction.

### The *Sophophora* Subgenus and Closely Related Taxa

We found that the *obscura*–*melanogaster* clade is the sister group of the lineages formed by the Neotropical *saltans* and *willistoni* groups, and the *Lordiphosa* genus (bootstrap percentage [BP]=73) (fig. 2A and supplementary fig. S3, Supplementary Material online). Thus, our study recovers the relationship between the groups of the *Sophophora* subgenus (Gao et al. 2011; Russo et al. 2013; Yassin 2013) and supports the paraphyletic status of *Sophophora* regarding *Lordiphosa* (Katoh et al. 2000). However, we noted substantial changes within the topology presented for the *melanogaster* species group. The original description of *Drosophila oshimai* noted a likeness to *Drosophila unipectinata*, thus classifying *D. oshimai* into the *suzukii* species subgroup (Choo and Nakamura 1973). The phylogenetic tree we obtained does not support this classification (fig. 2*A*). It rather defines *D. oshimai* as the representative of a new subgroup (Bayesian posterior probability [PP]=1, BP=96) that diverged immediately after the split of the *montium* group. The position of *D. oshimai* therefore challenges the monophyly of the *suzukii* subgroup. Interestingly, the paraphyly of the *suzukii* subgroup has also been suggested in previous studies (Lewis et al. 2005; Russo et al. 2013). Another interesting case is the positioning of the *denticulata* subgroup that has never been tested before. Our analysis convincingly places its representative species *Drosophila denticulata* as the fourth subgroup to branch off within the *melanogaster* group (PP=1, BP=82). Last, the topology within the *montium* group drastically differs from the most recent published phylogeny (Conner et al. 2021). Despite substantial sampling in the subgenus *Sophophora*, our study would benefit from the addition of representatives of the *dentissima*, *dispar*, *fima*, *populi*, *setifemur* groups, as well as the genus *Zapriothrica*, to draw a more complete picture of the relationships within *Sophophora*.

The genus *Collessia* comprises five described species that can be found in Australia, Japan, and Sri Lanka, but its phylogenetic status was so far quite ambiguous (Okada 1967, 1988; Bock 1982). In addition, Grimaldi (1990) proposed that *Tambourella ornata* should belong to the genus *Collessia*. These two genera are similar in the wing venation and pigmentation pattern (Okada 1984).

Our phylogenetic analysis identifies *Collessia* as sister group to the species *Hirtodrosophila duncani* (PP=1, BP=100). Interestingly, this branching is also supported by morphological similarities shared between the genera *Collessia* and *Hirtodrosophila*. The species *Collessia kirishimana* and *Collessia hiharai* were indeed initially described as *Hirtodrosophila* species (Okada 1967) but later assigned to the genus *Collessia* (Okada 1984), based on the similarity in wing coloration with *Collessia superba*. However, the affiliation of *Collessia kirishimana* to *Collessia* would require further investigations. The species *H. duncani* is morphologically disparate for *Hirtodrosophila* and might be removed from this genus in the future (Grimaldi 2018). The clade *Collessia–H. duncani* is sister to the *Sophophora*–*Lordiphosa* lineage in the ML inference (BP=100) but to the Neotropical *Sophophora*–*Lordiphosa* clade in the Bayesian inference (PP=0.92).

### The Early Lineage of *Microdrosophila* and *Dorsilopha*

Within the tribe Drosophilini, all the remaining taxa (composite taxa+ungrouped species) other than those of the *Sophophora*–*Lordiphosa* and *Collessia–H. duncani* lineage form a large clade (PP=1, BP=100). Within this clade, the genus *Microdrosophila*, the subgenus *Dorsilopha*, and *Drosophila ponera* group into a lineage (PP=0.97, BP=82) that appears as an early offshoot in our composite tree (fig. 2), reminiscent of the placement of *Dorsilopha* found in Yassin (2013). It is nevertheless noteworthy that the placement of the *Dorsilopha*+*Microdrosophila* clade differs in our supermatrix tree (fig. 1) and resembles the placement of *Microdrosophila* in Yassin (2013). In spite of scarce genomic data, we added the genus *Styloptera* which has been previously found close to the genus *Dorsilopha* (Yassin 2013). The position of *Styloptera* varies according to the analysis (supplementary fig. S9 and tree files, Supplementary Material online) without grouping with *Dorsilopha*. Generating genomic data for the genus *Styloptera* will be necessary to unambiguously place this genus. *Drosophila ponera* is an enigmatic species collected in La Réunion (David and Tsacas 1975), whose phylogenetic position has never or rarely been investigated. In spite of morphological similarities with the *quinaria* group, the authors suggested to keep *D. ponera* as ungrouped with respect to a divergent number of respiratory egg filaments (David and Tsacas 1975). To our knowledge, our study is the first attempt to phylogenetically position this species. We found that *D. ponera* groups with the *Dorsilopha* subgenus (PP=0.99, BP=75) within this early-diverging lineage.

### The Hawaiian Drosophilid Clade and the *Siphlodora* Subgenus

The endemic Hawaiian Drosophilidae contain approximately 1,000 species that split into the genera *Idiomyia* (or Hawaiian *Drosophila* according to Grimaldi [1990]) and the genus *Scaptomyza* (O’Grady et al. 2009). Generally considered as sister to the *Siphlodora* subgenus (Robe, Loreto, et al. 2010; Russo et al. 2013; Yassin 2013), these lineages represent a remarkable framework to investigate evolutionary radiation and subsequent diversification of morphology (Stark and O’Grady 2010), pigmentation (Edwards et al. 2007), ecology (Magnacca et al. 2008), and behavior (Kaneshiro 2001). Although the relationships within the *Siphlodora* clade are generally in agreement with previous studies (Tatarenkov et al. 2001; Robe et al. 2010; Russo et al. 2013; Yassin 2013), its sister clade does not seem to be restricted to the Hawaiian Drosophilidae. In fact, according to our phylogenies, it also includes at least four other species of the genus *Drosophila* (fig. 2*A* and supplementary fig. S3 and tree files, Supplementary Material online). We propose that this broader clade, rather than the Hawaiian clade sensu stricto, should be seen as a major lineage of Drosophilidae.

This broader clade is strongly supported (PP=1, BP=100) and divided into two subclades, one comprises the genera *Idiomyia* and *Scaptomyza* (PP=0.99, BP=97) and the other includes *Drosophila annulipes*, *Drosophila adamsi*, *Drosophila maculinotata*, and *Drosophila nigrosparsa* (PP=0.99, BP=75). The latter subclade, also suggested by Katoh et al. (2007) and Russo et al. (2013), is interesting with respect to the origin of Hawaiian drosophilids. Of the four component species, *D. annulipes* was originally described as a member of the subgenus *Spinulophila*, which was synonymized with *Drosophila* and currently corresponds to the *immigrans* group, although Wakahama et al. (1983) and Zhang and Toda (1992) cast doubt on its systematic position. The fact that *D. annulipes* does not belong to the immigrans species group implies that the subgenus *Drosophila* is paraphyletic rather than polyphyletic. As for *D*. *adamsi*, Da Lage et al. (2007) suggested it may be close to the *Idiomyia*–*Scaptomyza* clade, which is supported by our analyses. On the other hand, Prigent et al. (2013) based on morphological characters and Prigent et al. (2017) based on DNA barcoding have proposed that *D. adamsi* defines a new species group along with *Drosophila acanthomera* and an undescribed species. *Drosophila adamsi* resembles *D*. *annulipes* in the body color pattern (fig. 2*F*, *E*, and *H*), suggesting their close relationship: Adams (1905) described, “mesonotum with five longitudinal, brown vittae, the central one broader than the others and divided longitudinally by a hair-like line, …; scutellum yellow, with two sublateral, brownish lines, …; pleurae with three longitudinal brownish lines,” for *Drosophila quadrimaculata*Adams, 1905, which is a homonym of *Drosophila quadrimaculata* Walker, 1856 and has been replaced with the new specific epithet “*adamsi*” by Wheeler (1959). Another species, *D*. *nigrosparsa*, belongs to the *nigrosparsa* species group, along with *D*. *secunda*, *D*. *subarctica*, and *D*. *vireni* (Bächli et al. 2004). Moreover, Máca (1992) pointed out the close relatedness of *D*. *maculinotata* to the *nigrosparsa* group. It is noteworthy that the *nigrosparsa* species group is thought to be basal to *Siphlodora* in regard to the morphology of male genitalia (Yassin 2013).

### The *Drosophila* Subgenus and Closely Related Taxa

Although general relationships within the *Drosophila* subgenus closely resemble those recovered by previous studies (Hatadani et al. 2009; Robe et al. 2010; Robe et al. 2010; Izumitani et al. 2016), there are some outstanding results related to other genera or poorly studied *Drosophila* species.

*Samoaia* is a small genus of seven described species endemic to the Samoan Archipelago (Malloch 1934; Wheeler and Kambysellis 1966), particularly studied for their body and wing pigmentation (Dufour et al. 2020). In our analysis, the genus *Samoaia* is found to group with the *quadrilineata* species subgroup of the *immigrans* group. This result is similar to conclusions formulated by some previous studies (Tatarenkov et al. 2001; Robe et al. 2010; Yassin et al. 2010; Yassin 2013), but differs from other published phylogenies in which *Samoaia* is sister to most other lineages in the subgenus *Drosophila* (Russo et al. 2013). It is noteworthy that our sampling is the most substantial with four species of *Samoaia*.

The two African species *Drosophila pruinosa* and *Drosophila pachneissa*, which were assigned to the *loiciana* species complex because of shared characters such as a glaucous-silvery frons and rod-shaped surstylus (Tsacas 2002), are placed together with the *immigrans* group (PP=1, BP=94). In previous large-scale analyses, *D. pruinosa* was suggested to group with *Drosophila sternopleuralis* into the sister clade of the *immigrans* group (Da Lage et al. 2007; Russo et al. 2013).

Among other controversial issues, the phylogenetic position of *Drosophila aracea* was previously found to markedly change according to the phylogenetic reconstruction methods (Da Lage et al. 2007). This anthophilic species lives in Central America (Heed and Wheeler 1957). Its name comes from the behavior of females that lay eggs on the spadix of plants in the family Araceae (Heed and Wheeler 1957; Tsacas and Chassagnard 1992). Our analysis places *D. aracea* as the sister taxon of the *bizonata*–*testacea* clade with high confidence (PP=1, BP=85). No occurrence of flower-breeding behavior has been reported in the *bizonata*–*testacea* clade, reinforcing the idea that *D. aracea* might have recently evolved from a generalist ancestor (Tsacas and Chassagnard 1992).

### The *Zygothrica* Genus Group

The fungus-associated genera *Hirtodrosophila*, *Mycodrosophila*, *Paraliodrosophila*, *Paramycodrosophila*, and *Zygothrica* contain 449 identified species (DrosWLD-Species 2021; https://bioinfo.museum.hokudai.ac.jp/db/index.php; last accessed June 29, 2021) and have been associated with the *Zygothrica* genus group (Grimaldi 1990). Although the *Zygothrica* genus group was recurrently recovered as paraphyletic (Da Lage et al. 2007; Van Der Linde et al. 2010; Russo et al. 2013; Yassin 2013), two recent studies suggest, on the contrary, its monophyly (Gautério et al. 2020; Zhang et al. 2021). Our study does not support the monophyly of the *Zygothrica* genus group in virtue of the polyphyletic status of *Hirtodrosophila* and *Zygothrica*: some representatives (e.g., *H. duncani*) cluster with *Collessia*, whereas others (e.g., *Hirtodrosophila* IV and *Zygothrica* II) appear closely related to the genera *Dichaetophora* and *Mulgravea*. Furthermore, the placement of the *Zygothrica* genus group recovered in our study also differs from some previous estimates. In fact, the broadly defined *Zygothrica* genus group, which includes *Dichaetophora* and *Mulgravea* (PP=0.95, BP=64), appears as sister to the clade composed of the subgenus *Drosophila* and the *Hypselothyrea*/*Liodrosophila*+*Sphaerogastrella*+*Zaprionus* clade (PP=1, BP=56) (fig. 2*A* and supplementary fig. S3, Supplementary Material online). This placement is similar to the ones obtained in different studies (Van Der Linde et al. 2010; Russo et al. 2013), but contrasts with the close relationship of the *Zygothrica* genus group to the subgenus *Siphlodora*+*Idiomyia*/*Scaptomyza* proposed in two recent studies (Gautério et al. 2020; Zhang et al. 2021). Given the moderate bootstrap value, the exact status of the *Zygothrica* genus group remains as an open question.

Furthermore, within the superclade of the broadly defined *Zygothrica* genus group (figs. 1 and 2*A*), the genus *Hirtodrosophila* is paraphyletic and split into four independent lineages, reinforcing previous suggestions based on multilocus approaches (Van Der Linde et al. 2010; Gautério et al. 2020; Zhang et al. 2021). This also occurred with the genus *Zygothrica*, which split into two independent clades (fig. 2*A*). The *leptorostra* subgroup (*Zygothrica* II) clusters with the subgroup *Hirtodrosophila* IV (PP=1, BP=100), whereas the *Zygothrica* I subgroup clusters with the species *Hirtodrosophila levigata* (PP=0.99, BP=98).

### DrosoPhyla: A Powerful Tool for Systematics

Besides bringing an updated and improved phylogenetic framework to Drosophilidae, our approach also addresses several questions that were previously unassessed or controversial at the genus, subgenus, group, or species level. We are therefore confident that it may become a powerful tool for future drosophilid systematics. According to diversity surveys (O’Grady and DeSalle 2018), ∼25% of drosophilid species remain to be discovered, potentially a thousand species to place in the tree of Drosophilidae. Although whole-genome sequencing is becoming widespread, newly discovered species often come down to a few specimens pinned or stored in ethanol—nonoptimal conditions for subsequent genome sequencing and whole-genome studies (Korlević et al. 2021). An alternative promising approach to PCR is exome capture using baits to hybridize to genomic regions of interest, which has been used with other insects (Branstetter et al. 2017). Nevertheless, based on a few short genomic markers, our approach is compatible with taxonomic work, and gives good resolution.

## Materials and Methods

### Taxon Sampling

The species used in this study were sampled from different locations throughout the world (supplementary table S1, Supplementary Material online). The specimens were field-collected by the authors, purchased from the National Drosophila Species Stock Center (http://blogs.cornell.edu/drosophila/; last accessed January 2021) and the Kyoto Stock Center (https://kyotofly.kit.jp/cgi-bin/stocks/index.cgi; last accessed January 2021), or obtained from colleagues. Individual flies were preserved in 100% ethanol and identified based on morphological characters.

### Data Collection

Ten genomic markers were amplified by PCR using degenerate primers developed for the present study (table 2). Genomic DNA was extracted from a single adult fly as follows: the fly was placed in a 0.5-ml tube and mashed in 50 µl of squishing buffer (Tris–HCl pH = 8.2 10 mM, EDTA 1 mM, NaCl 25 mM, proteinase K 200 µg/ml) for 20–30 s, the mix was incubated at 37 °C for 30 min, then the proteinase K was inactivated by heating at 95 °C for 1–2 min. A volume of 1 µl was used as template for PCR amplification. Nucleotide sequences were also retrieved from the NCBI database for the five nuclear markers *28S ribosomal RNA* (*28S*), *alcohol dehydrogenase* (*Adh*), *glycerol-3-phosphate dehydrogenase* (*Gpdh*), *superoxide dismutase* (*Sod*), *xanthine dehydrogenase* (*Xdh*), and the two mitochondrial markers *cytochrome oxidase subunit 1* (*COI*) and *cytochrome oxidase subunit 2* (*COII*). The sequences reported in this article have been deposited in GenBank under specific accession numbers: *Amyrel* (MW392482–MW392524), *Ddc* (MW403139–MW403307), *Dll* (MW403308–MW403483), *eb* (MW415022–MW415267), *en* (MW418945–MW419079), *eve* (MW425034–MW425273), *hh* (MW385549–MW385782), *Notum* (MW429853–MW430003), *ptc* (MW442160–MW442361), and *wg* (MW392301–MW392481).

**Table 2:** List of PCR Primers Used in This Study

Genomic Locus	Primer	Primer Sequence (5′–3′)	Annealing (°C)	Size (bp)	References
*Amyrel*	zone2bis	GTAAATNGGNNCCACGCGAAG	53	1,000	Da Lage et al. (2007)
	relrev+	GTTCCCCAGCTCTGCAGCC			
	reludir	TGGATGCNGCCAAGCACATGGC		1,000	
	relavbis	GCATTTGTACCGTTTGTGTCGTTATCG			
*Distal-less*	dll-F	TGATACCAATACTGSGGCACATA	56	600	This study
	dll-R	ATGATGAARGCMGCTCAGGG			
*Dopa decarboxylase*	ddc-F	TTCCASGAGTACTCCATGTCCTCG	58	1,200	This study
	ddc-R	GGCAGGATGTKATGAAGGACATTGAG			
*ebony*	eb-F	CCCATSACCTCKGTGGAGCCGTA	59	900	This study
	eb-R	CTGCATCGCATCTTYGAGGAGCA			
*engrailed*	en-F	AATCAGCGCCCAGTCCACCAG	65	1,500	This study
	en-R	GCCACATCTCGTTCTTGCCGC			
*even-skipped*	eve-F	TGCCTVTCCAGTCCRGAYAACTC	55	1,000	This study
	eve-R	TACGCCTCAGTCTTGTAGGG			
*hedgehog*	hh-F	ACCTTGTABARGGCATTGGCATACCA	56	600	This study
	hh-R	ATCGGWGATCGDGTGCTRAGCATG			
*Notum*	not-F	TGGAACTAYATHCAYGADATGGGCGG	56	800	This study
	not-R	GAGCAGYTCVAGRAADCGCATCTC			
*patched*	ptc-F1	ACCCAGCTGCGCATSAGRAAGG	54	600	This study
	ptc-F2	ACCCAGCTGCGCATSAGRAACG			
	ptc-R	GCTGACGGCSGCSTATGCGG			
*wingless*	wg-F	AGCACGTYCARGCRGAGATGCG	58	400	This study
	wg-R	ACTGTTKGGCGAYGGCATRTTGGG			

### Phylogenetic Reconstruction

Alignments for each individual gene were generated using MAFFT 7.45 (Katoh and Standley 2013) assuming a gap opening penalty of 1.53 and other default parameters (no offset and extra round of refinement). Unreliably aligned positions were excluded using trimAl with parameters -gt 0.5 and -st 0.001 (Capella-Gutiérrez et al. 2009). The possible contamination status was verified by inferring independent trees for each gene using RAxML 8.2.4 under the GTR+Γ_4_ model (Stamatakis 2014). Thus, any sequence leading to the suspicious placement of a taxonomically well-assigned species, in terms of both topology and bootstrap value, was removed from the data set. Moreover, almost identical sequences leading to very short tree branches were carefully examined and excluded if involving nonclosely related taxa. In-house Python scripts were used to concatenate the aligned and filtered sequences, and the resulting data set was used for phylogenetic reconstruction. Maximum-likelihood (ML) searches were performed using IQ-TREE 2.0.6 (Minh, Schmidt, et al. 2020) under the GTR model, with the FreeRate model of rate heterogeneity across sites with four categories, and ML estimation of base frequencies from the data (GTR+R+FO). The edge-linked proportional partition model was used with one partition for each gene.

### Composite Taxa

This strategy started from clustering the species by unambiguous monophyletic genera, groups, or subgroups identified in the 704-taxon analysis. After this, the least diverging sequence or species recovered for each taxonomic unit for each marker was selected to ultimately yield a unique composite taxon by concatenation. The composite matrix was also used for conducting ML and Bayesian phylogenetic inference using IQ-TREE under a partitioned GTR+R+FO model (parameters: -m GTR+FO+R -B 1000 -bnni -p), and PhyloBayes under a GTR+Γ model (parameters: -ncat 1 -gtr) (Lartillot et al. 2009), respectively.

### Saturation and Concordance Analysis

For each marker gene, the saturation was computed by performing a simple linear regression of the percent identity for each pair of taxa (observed distance) onto the ML patristic distance (inferred distance) (Philippe et al. 1994) estimated using the ETE 3 library (Huerta-Cepas et al. 2016). We also calculated per gene and per site concordance factors using IQ-TREE under the GTR+R+FO model as recently described (Minh, Hahn, et al. 2020). We also applied ASTRAL to estimate species tree from individual species tree, using default parameters and the same input single gene trees (Zhang et al. 2018).

## Supplementary Material

Supplementary data are available at *Genome Biology and Evolution* online.

## Supplementary Material

evab179_Supplementary_Data

## Data Availability

The data underlying this article are available on Zenodo (10.5281/zenodo.5091961).
