# Supplementary material for: DrosoPhyla: Resources for Drosophilid Phylogeny and Systematics
Source: Genome Biol Evol. 2021 Aug 3;13(8):evab179. doi: 10.1093/gbe/evab179 (PMC8382681; doi:10.1093/gbe/evab179)
Supplement: evab179_Supplementary_Data [file evab179_supplementary_data.zip › Supplementary data.pdf]

Supplementary Figure and Table Legends

Figure S1. Phylogram of the 204-taxon analysis. IQ-TREE maximum-likelihood analyses were conducted using the GTR+R+FO model. Support values obtained after 100 bootstrap replicates are shown for all branches. Scale bar indicates the number of changes per site.

Figure S2. Phylogram of the 204-taxon analysis. PhyloBayes Bayesian analyses were conducted using the GTR+G model. Bayesian posterior probabilities are shown for all branches. Scale bar indicates the number of changes per site.

Figure S3. Phylogram of the 83-taxon analyses. (Left) IQ-TREE maximum-likelihood analyses were conducted using the GTR+R+FO model. Support values obtained after 100 bootstrap replicates are shown for all branches. Scale bar indicates the number of changes per site. (Right) PhyloBayes Bayesian analyses were conducted using the GTR+G model. Bayesian posterior probabilities are shown for all branches. Scale bar indicates the number of changes per site.

Figure S4. Comparison of support values between the non-composite and composite maximum-likelihood trees. All support values were obtained after 100 bootstrap replicates. The first value refers to the composite approach (83 taxa), and the second value in parentheses refers to the non-composite approach (704 taxa).

Figure S5. Phylogram of the 83-taxon ASTRAL analysis. Branch support values measure the support for a quadripartition (the four cluster around a branch) and not the bipartition, as is commonly done. Scale bar indicates the number of changes per site.

Figure S6. The impact of marker sampling on the tree topology. The composite tree was built on 17 different datasets that correspond to the whole dataset minus one marker sequentially removed. The changes in relation to the ML composite tree depicted in Figure 2 are shown in red. Scale bar indicates the number of changes per site.

Figure S7. Mutational saturation of the 17 phylogenetic markers. The x-axis indicates the distance inferred from the ML composite tree, whereas the y-axis indicates the observed distance between two taxa. The slope of the red line is an indicator of the saturation level, low values meaning high saturation. The black line corresponds to the absence of multiple substitutions.

Figure S8. Phylogram of the Steganinae subfamily. This ML tree was built on a dataset that includes 164 steganine taxa. IQ-TREE maximum-likelihood analysis was conducted under the GTR+R+FO model. Support values obtained after 100 bootstrap replicates are shown for selected branches (all the support values are available online). Scale bar indicates the number of changes per site.

Figure S9. Addition of missing taxa with scarce genomic data to the composite tree. We added the published sequences of the genera *Jeannelopsis*, *Lissocephala*, *Neotanygastrella*, *Phorticella*, *Styloptera* (Yassin 2013), the subgenus *Dudaica* (Katoh et al. 2018), and several *Hirtodrosophila* and *Zygothrica* species (Gautério et al. 2020) to our 83-taxon composite dataset to draw a more comprehensive picture of the Drosophilinae, especially the tribe *Colocasiomyini*.

Table S1. Taxon sampling and presence/absence of markers per taxon. Markers generated in this study are indicated in black, markers retrieved from GenBank are indicated in grey, missing data are indicated in white.

Figure S1

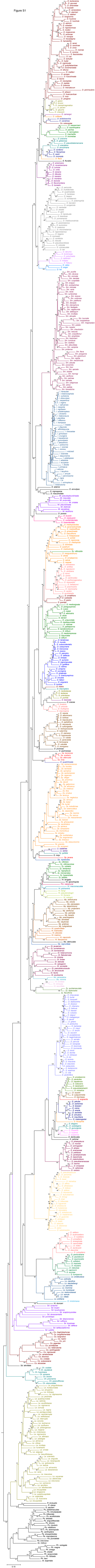

Figure S2

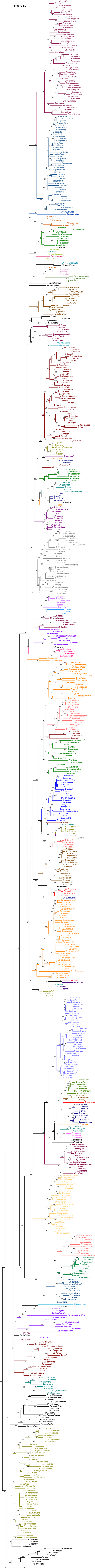

Figure S3

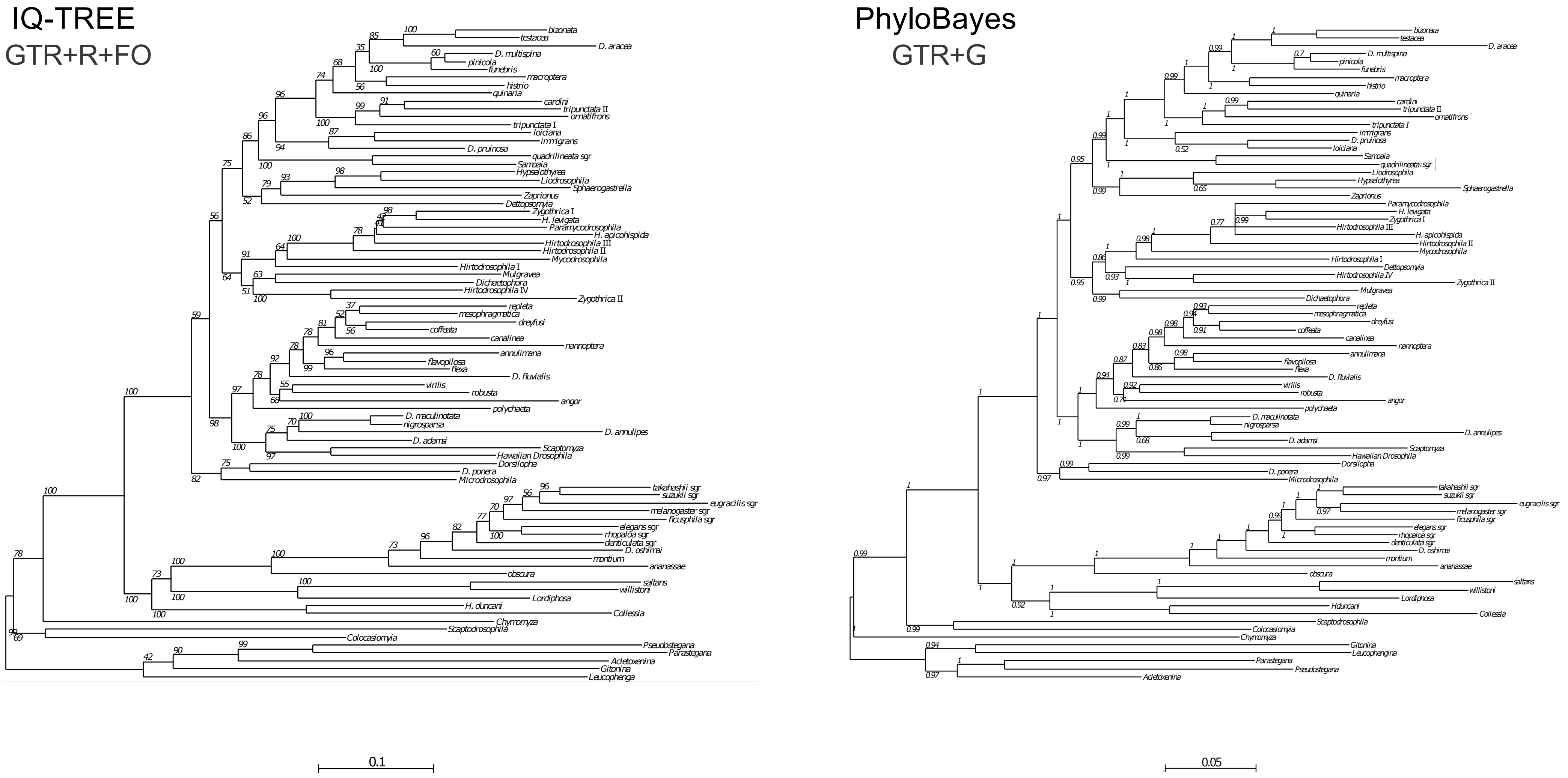
IQ-TREE
GTR+R+FO
bizonata
testacea
D. aracea
D. multispina
pinicola
funebris
macroptera
histrio
quinaria
cardini
tripunctata II
ornatifrons
tripunctata I
loiciana
immigrans
D. pruinosa
quadrilineata sgr
Samoaia
Hypselothyrea
Liodrosophila
Sphaerogastrella
Zaprionus
Dettopsomyia
Zygothrica I
H. levigata
Paramycodrosophila
H. apicohispida
Hirtodrosophila III
Hirtodrosophila II
Mycodrosophila
Hirtodrosophila I
Mulgravea
Dichaetophora
Hirtodrosophila IV
Zygothrica II
repleta
mesophragmatica
dreyfusi
coffeata
canalinea
nannoptera
annulimana
flavopilosa
flexa
D. fluvialis
virilis
robusta
angor
polychaeta
D. maculinotata
nigrosparsa
D. annulipes
D. adamsi
Scaptomyza
Hawaiian Drosophila
Dorsilopha
D. ponera
Microdrosophila
takahashii sgr
suzukii sgr
eugracilis sgr
melanogaster sgr
ficusphila sgr
elegans sgr
rhopaloa sgr
denticulata sgr
D. oshimai
montium
ananassae
obscura
saltans
willistoni
Lordiphosa
H. duncani
Collessia
Chymomyza
Scaptodrosophila
Colocasiomyia
Pseudostegana
Parastegana
Acletoxenina
Gitonina
Leucophenga
0.1
PhyloBayes
GTR+G
bizonata
testacea
D. aracea
D. multispina
pinicola
funebris
macroptera
histrio
quinaria
cardini
tripunctata II
ornatifrons
tripunctata I
immigrans
D. pruinosa
loiciana
Samoaia
quadrilineata sgr
Liodrosophila
Hypselothyrea
Sphaerogastrella
Zaprionus
Paramycodrosophila
H. levigata
Zygothrica I
Hirtodrosophila III
H. apicohispida
Hirtodrosophila II
Mycodrosophila
Hirtodrosophila I
Dettopsomyia
Hirtodrosophila IV
Zygothrica II
Mulgravea
Dichaetophora
repleta
mesophragmatica
dreyfusi
coffeata
canalinea
nannoptera
annulimana
flavopilosa
flexa
D. fluvialis
virilis
robusta
angor
polychaeta
D. maculinotata
nigrosparsa
D. adamsi
D. annulipes
Scaptomyza
Hawaiian Drosophila
Dorsilopha
D. ponera
Microdrosophila
takahashii sgr
suzukii sgr
eugracilis sgr
melanogaster sgr
ficusphila sgr
elegans sgr
rhopaloa sgr
denticulata sgr
D. oshimai
montium
ananassae
obscura
saltans
willistoni
Lordiphosa
Hduncani
Collessia
Scaptodrosophila
Colocasiomyia
Chymomyza
Gitonina
Leucophengina
Parastegana
Pseudostegana
Acletoxenina
0.05

Figure S4

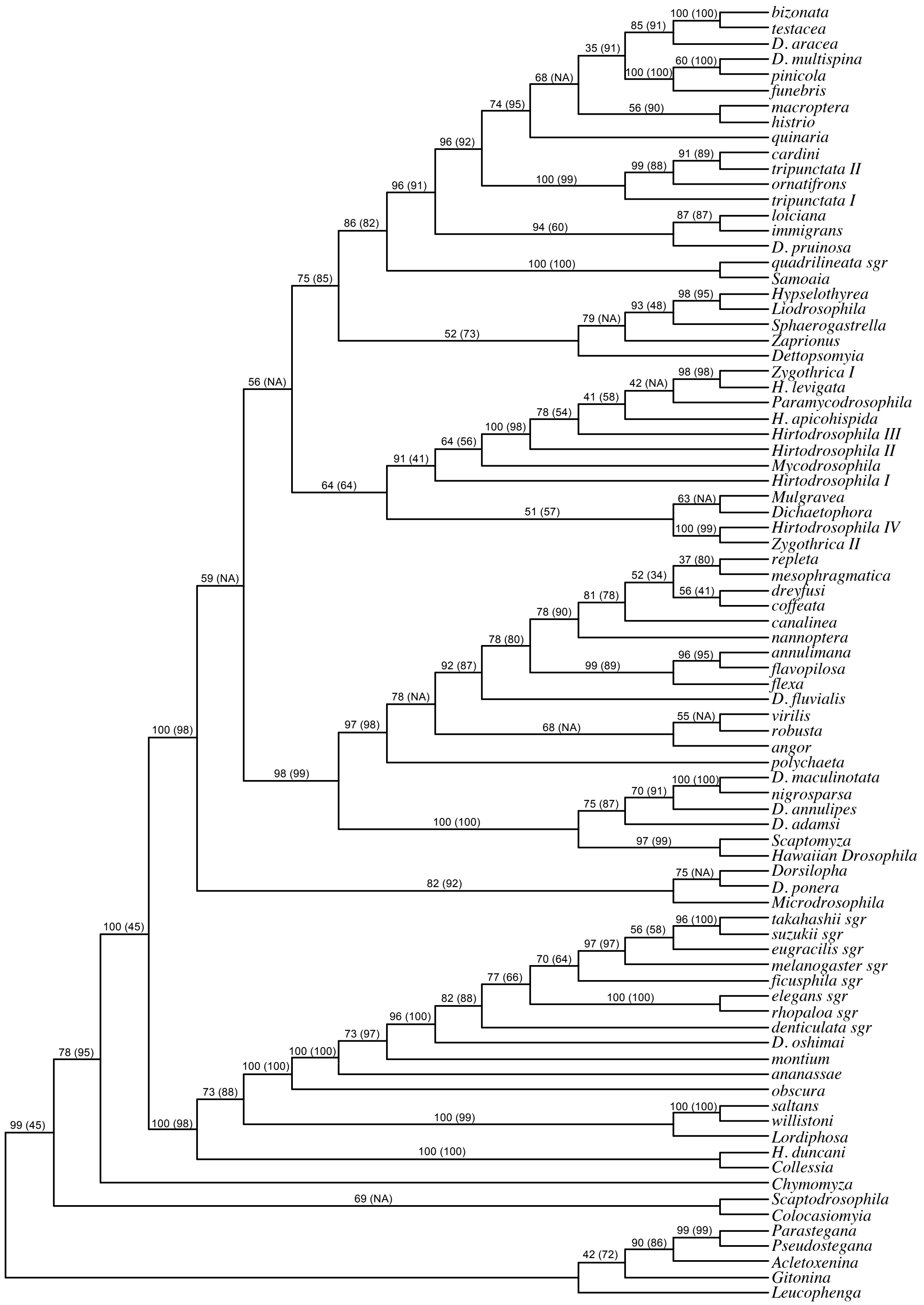

bizonata
testacea
D. aracea
D. multispina
pinicola
funebris
macroptera
histrio
quinaria
cardini
tripunctata II
ornatifrons
tripunctata I
loiciana
immigrans
D. pruinosa
quadrilineata sgr
Samoaia
Hypselothyrea
Liodrosophila
Sphaerogastrella
Zaprionus
Dettopsomyia
Zygothrica I
H. levigata
Paramycodrosophila
H. apicohispida
Hirtodrosophila III
Hirtodrosophila II
Mycodrosophila
Hirtodrosophila I
Mulgravea
Dichaetophora
Hirtodrosophila IV
Zygothrica II
repleta
mesophragmatica
dreyfusi
coffeata
canalinea
nannoptera
annulimana
flavopilosa
flexa
D. fluvialis
virilis
robusta
angor
polychaeta
D. maculinotata
nigrosparsa
D. annulipes
D. adamsi
Scaptomyza
Hawaiian Drosophila
Dorsilopha
D. ponera
Microdrosophila
takahashii sgr
suzukii sgr
eugracilis sgr
melanogaster sgr
ficusphila sgr
elegans sgr
rhopaloa sgr
denticulata sgr
D. oshimai
montium
ananassae
obscura
saltans
willistoni
Lordiphosa
H. duncani
Collessia
Chymomyza
Scaptodrosophila
Colocasiomyia
Parastegana
Pseudostegana
Acletoxenina
Gitonina
Leucophenga
100 (100)
85 (91)
35 (91)
60 (100)
100 (100)
68 (NA)
56 (90)
74 (95)
96 (92)
91 (89)
99 (88)
100 (99)
96 (91)
87 (87)
94 (60)
86 (82)
100 (100)
75 (85)
98 (95)
93 (48)
79 (NA)
52 (73)
56 (NA)
98 (98)
42 (NA)
41 (58)
78 (54)
100 (98)
64 (56)
91 (41)
64 (64)
63 (NA)
51 (57)
100 (99)
59 (NA)
37 (80)
52 (34)
56 (41)
81 (78)
78 (90)
78 (80)
96 (95)
99 (89)
92 (87)
78 (NA)
55 (NA)
68 (NA)
97 (98)
100 (98)
98 (99)
100 (100)
70 (91)
75 (87)
100 (100)
97 (99)
75 (NA)
82 (92)
100 (45)
96 (100)
56 (58)
97 (97)
70 (64)
77 (66)
100 (100)
82 (88)
96 (100)
73 (97)
100 (100)
100 (100)
78 (95)
73 (88)
100 (100)
100 (99)
99 (45)
100 (98)
100 (100)
69 (NA)
99 (99)
90 (86)
42 (72)

Figure S5

ASTRAL

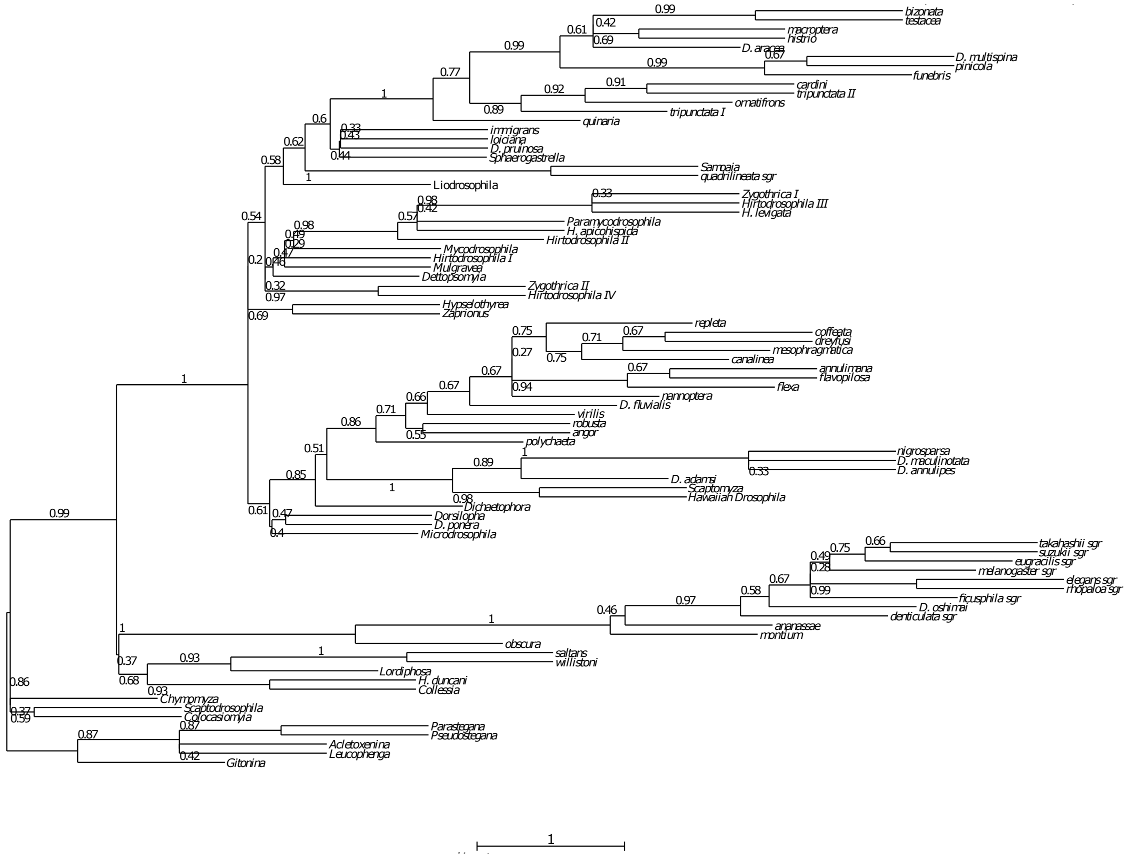

Figure S6

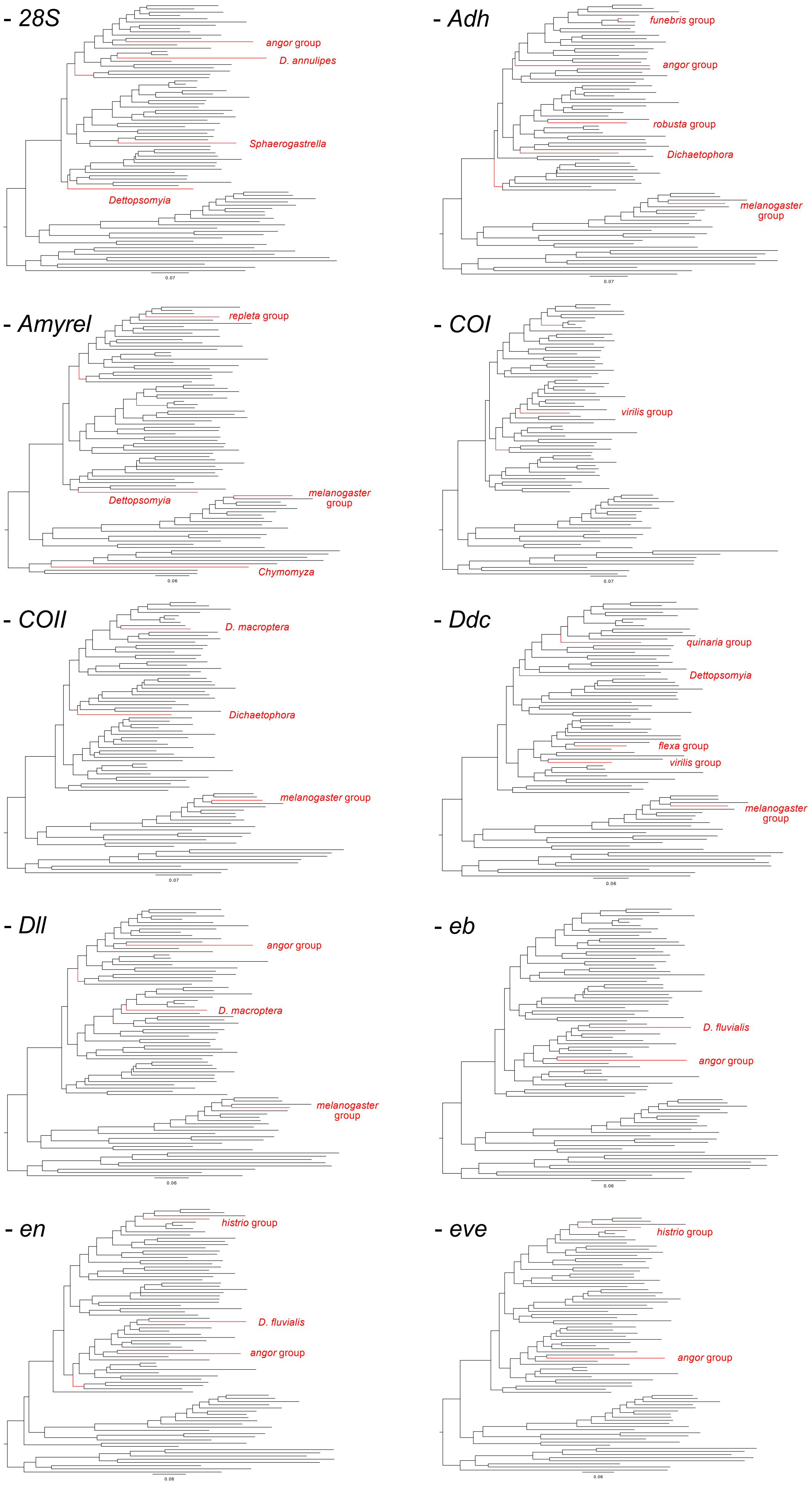

- 28S
angor group
D. annulipes
Sphaerogastrella
Dettopsomyia
- Adh
funebris group
angor group
robusta group
Dichaetophora
melanogaster group
- Amyrel
repleta group
Dettopsomyia
melanogaster group
Chymomyza
- COI
virilis group
- COII
D. macroptera
Dichaetophora
melanogaster group
- Ddc
quinaria group
Dettopsomyia
flexa group
virilis group
melanogaster group
- Dll
angor group
D. macroptera
melanogaster group
- eb
D. fluvialis
angor group
- en
histrio group
D. fluvialis
angor group
- eve
histrio group
angor group

Figure S7

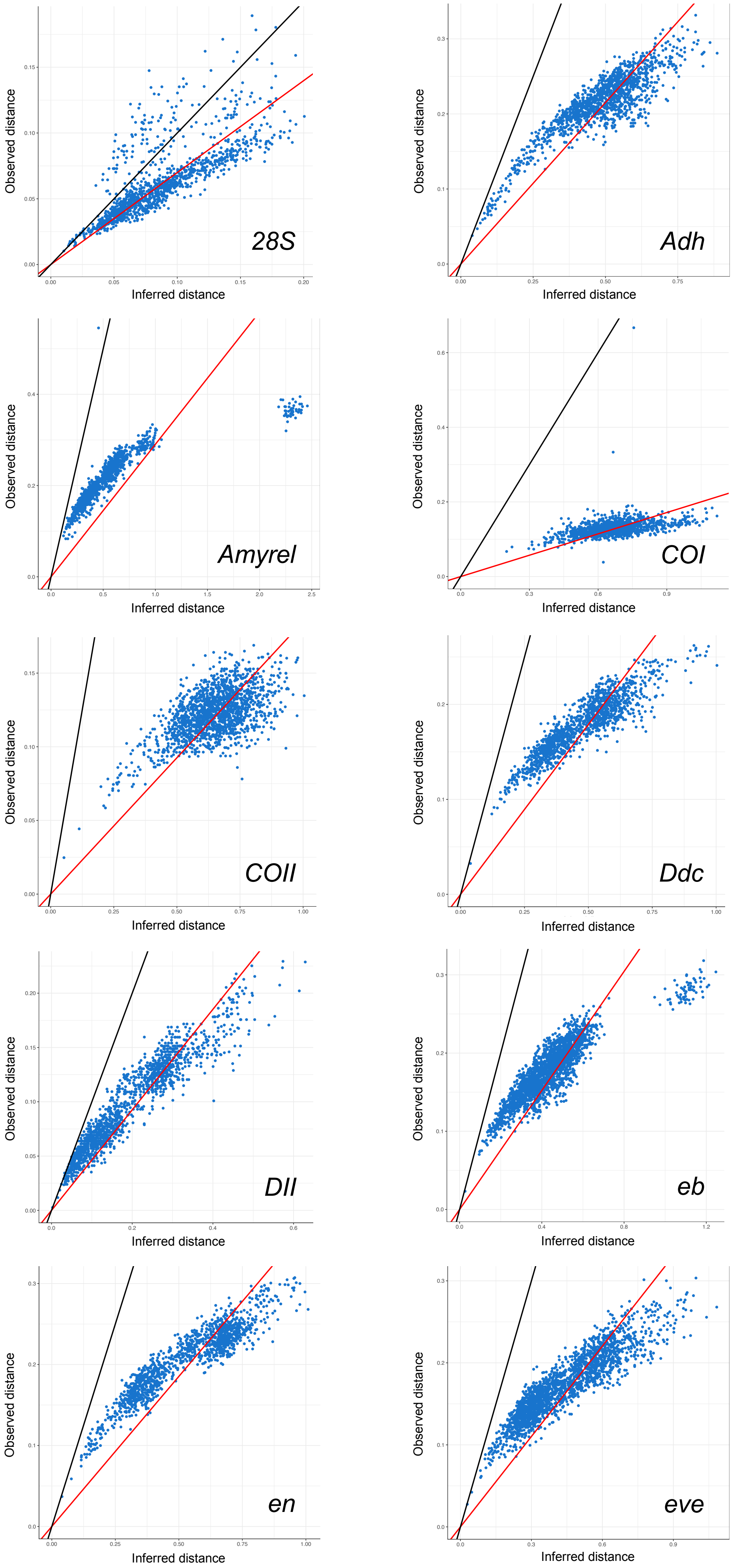

Observed distance
Inferred distance
28S
Adh
Amyrel
COI
COII
Ddc
Dll
eb
en
eve

Figure S8

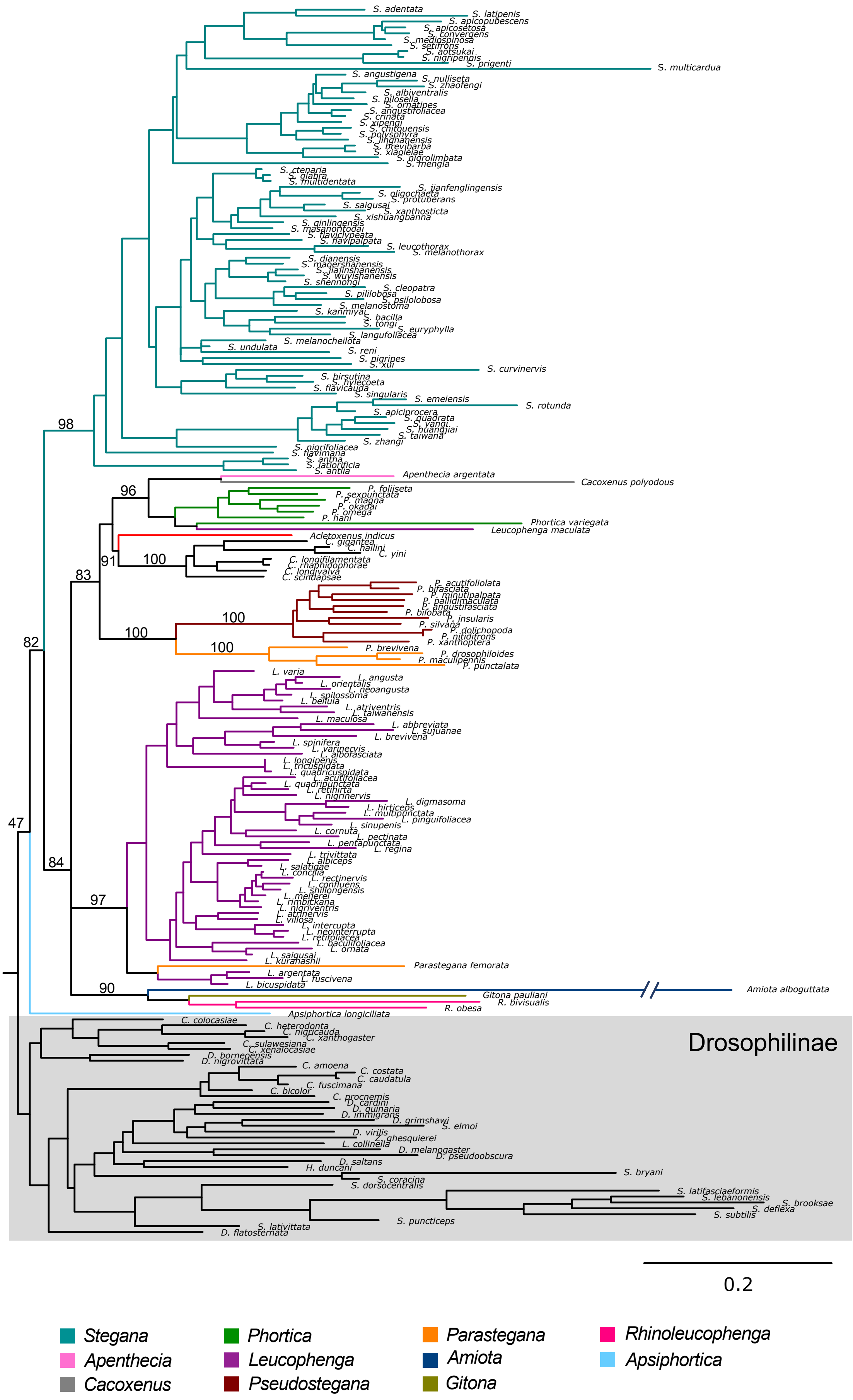

S. adentata
S. latipenis
S. apicopubescens
S. apicosetosa
S. convergens
S. mediospinosa
S. setifrons
S. aotsukai
S. nigripennis
S. prigenti
S. multicardua
S. angustigena
S. nulliseta
S. zhaofengi
S. albiventralis
S. pilosella
S. ornatipes
S. angustifoliacea
S. crinata
S. xipengi
S. chitouensis
S. polysphyra
S. jinghanensis
S. brevibarba
S. xiaoleiae
S. nigrolimbata
S. mengla
S. ctenaria
S. glabra
S. multidentata
S. jianfenglingensis
S. oligochaeta
S. protuberans
S. saigusai
S. xanthosticta
S. xishuangbanna
S. qinlingensis
S. masanoritodai
S. flaviclypeata
S. flavipalpata
S. leucothorax
S. melanothorax
S. dianensis
S. maoershanensis
S. jiajinshanensis
S. wuyishanensis
S. shennongi
S. cleopatra
S. pililobosa
S. psilolobosa
S. melanostoma
S. kanmiyai
S. bacilla
S. tongi
S. euryphylla
S. langufoliacea
S. melanocheilota
S. undulata
S. reni
S. nigripes
S. xui
S. curvinervis
S. hirsutina
S. hylecoeta
S. flavicauda
S. singularis
S. emeiensis
S. rotunda
S. apiciprocera
S. quadrata
S. yangi
S. huangjiai
S. taiwana
S. zhangi
S. nigrifoliacea
S. flavimana
S. antha
S. latiorificia
S. antlia
Apenthecia argentata
Cacoxenus polyodous
P. foliiseta
P. sexpunctata
P. magna
P. okadai
P. omega
P. hani
Phortica variegata
Leucophenga maculata
Acletoxenus indicus
C. gigantea
C. hailini
C. yini
C. longifilamentata
C. rhaphidophorae
C. longivalva
C. scindapsae
P. acutifoliolata
P. bifasciata
P. minutipalpata
P. pallidimaculata
P. angustifasciata
P. bilobata
P. insularis
P. silvana
P. dolichopoda
P. nitidifrons
P. xanthoptera
P. brevivena
P. drosophiloides
P. maculipennis
P. punctalata
L. varia
L. angusta
L. orientalis
L. neoangusta
L. spilossoma
L. bellula
L. atriventris
L. taiwanensis
L. maculosa
L. abbreviata
L. sujuanae
L. brevivena
L. spinifera
L. varinervis
L. albofasciata
L. longipenis
L. tricuspidata
L. quadricuspidata
L. acutifoliacea
L. quadripunctata
L. retihirta
L. nigrinervis
L. digmasoma
L. hirticeps
L. multipunctata
L. pinguifoliacea
L. sinupenis
L. cornuta
L. pectinata
L. pentapunctata
L. regina
L. trivittata
L. albiceps
L. salatigae
L. concilia
L. rectinervis
L. confluens
L. shillongensis
L. meijerei
L. rimbickana
L. nigriventris
L. atrinervis
L. villosa
L. interrupta
L. neointerrupta
L. retifoliacea
L. baculifoliacea
L. ornata
L. saigusai
L. kurahashii
Parastegana femorata
L. argentata
L. fuscivena
L. bicuspidata
Gitona pauliani
Amiota alboguttata
R. bivisualis
R. obesa
Apsiphortica longiciliata
C. colocasiae
C. heterodonta
C. nigricauda
C. xanthogaster
C. sulawesiana
C. xenalocasiae
D. borneensis
D. nigrovittata
C. amoena
C. costata
C. caudatula
C. fuscimana
C. bicolor
C. procnemis
D. cardini
D. quinaria
D. immigrans
D. grimshawi
S. elmoi
D. virilis
Z. ghesquierei
L. collinella
D. melanogaster
D. pseudoobscura
D. saltans
H. duncani
S. bryani
S. coracina
S. dorsocentralis
S. latifasciaeformis
S. lebanonensis
S. brooksae
S. deflexa
S. subtilis
S. puncticeps
S. lativittata
D. flatosternata
98
96
91
100
83
100
100
100
82
47
84
97
90
Drosophilinae
0.2
Stegana
Apenthecia
Cacoxenus
Phortica
Leucophenga
Pseudostegana
Parastegana
Amiota
Gitona
Rhinoleucophenga
Apsiphortica

Figure S9

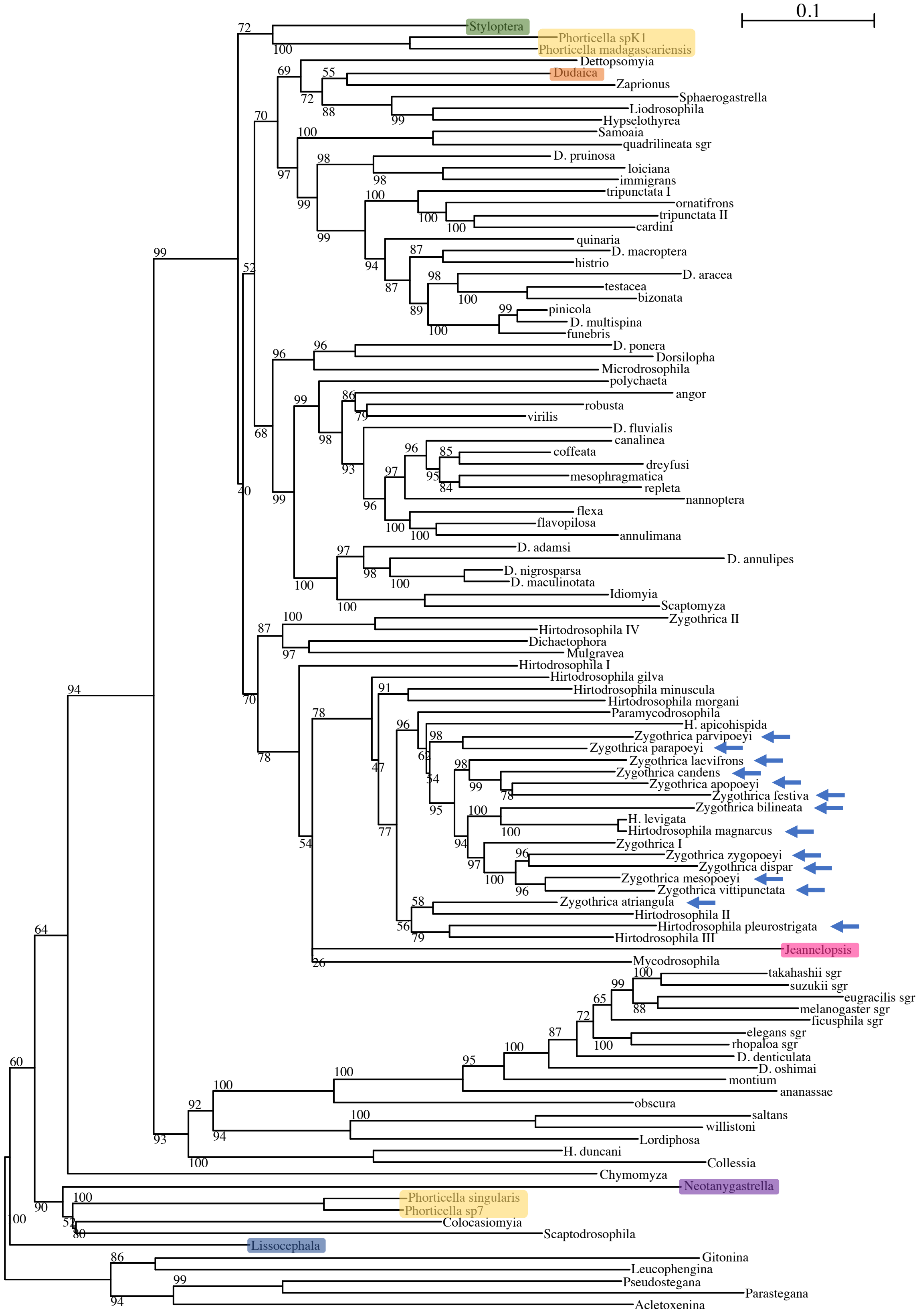
0.1
Styloptera
Phorticella spK1
Phorticella madagascariensis
Dettopsomyia
Dudaica
Zaprionus
Sphaerogastrella
Liodrosophila
Hypselothyrea
Samoaia
quadrilineata sgr
D. pruinosa
loiciana
immigrans
tripunctata I
ornatifrons
tripunctata II
cardini
quinaria
D. macroptera
histrio
D. aracea
testacea
bizonata
pinicola
D. multispina
funebris
D. ponera
Dorsilopha
Microdrosophila
polychaeta
angor
robusta
virilis
D. fluvialis
canalinea
coffeata
dreyfusi
mesophragmatica
repleta
nannoptera
flexa
flavopilosa
annulimana
D. adamsi
D. annulipes
D. nigrosparsa
D. maculinotata
Idiomyia
Scaptomyza
Zygothrica II
Hirtodrosophila IV
Dichaetophora
Mulgravea
Hirtodrosophila I
Hirtodrosophila gilva
Hirtodrosophila minuscula
Hirtodrosophila morgani
Paramycodrosophila
H. apicohispida
Zygothrica parvipoeyi
Zygothrica parapoeyi
Zygothrica laevifrons
Zygothrica candens
Zygothrica apopoeyi
Zygothrica festiva
Zygothrica bilineata
H. levigata
Hirtodrosophila magnarcus
Zygothrica I
Zygothrica zygopoeyi
Zygothrica dispar
Zygothrica mesopoeyi
Zygothrica vittipunctata
Zygothrica atriangula
Hirtodrosophila II
Hirtodrosophila pleurostrigata
Hirtodrosophila III
Jeannelopsis
Mycodrosophila
takahashii sgr
suzukii sgr
eugracilis sgr
melanogaster sgr
ficusphila sgr
elegans sgr
rhopaloa sgr
D. denticulata
D. oshimai
montium
ananassae
obscura
saltans
willistoni
Lordiphosa
H. duncani
Collessia
Chymomyza
Neotanygastrella
Phorticella singularis
Phorticella sp7
Colocasiomyia
Scaptodrosophila
Lissocephala
Gitonina
Leucophengina
Pseudostegana
Parastegana
Acletoxenina
